# Inertial measurement unit-based real-time feedback gait immediately changes gait parameters in older inpatients: a pilot study

**DOI:** 10.3389/fphys.2024.1384313

**Published:** 2024-08-06

**Authors:** Takasuke Miyazaki, Ryoji Kiyama, Yasufumi Takeshita, Daichi Shimose, Sota Araki, Hisanori Matsuura, Yuki Uto, Shobu Nakashima, Yuki Nakai, Masayuki Kawada

**Affiliations:** ^1^ Department of Orthopedic Surgery, Graduate School of Medical and Dental Sciences, Kagoshima University, Kagoshima, Japan; ^2^ Department of Physical Therapy, School of Health Sciences, Faculty of Medicine, Kagoshima University, Kagoshima, Japan; ^3^ Sports Science Area, Department of Mechanical Systems Engineering, Daiichi Institute of Technology, Kagoshima, Japan; ^4^ Course of Health Sciences, Graduate School of Health Sciences, Kagoshima University, Kagoshima, Japan; ^5^ Department of Rehabilitation, Tarumizu Municipal Medical Center, Tarumizu Central Hospital, Kagoshima, Japan

**Keywords:** clinical application, gait training, wearable sensor, gait analysis, propulsion

## Abstract

The effect of gait feedback training for older people remains unclear, and such training methods have not been adapted in clinical settings. This study aimed to examine whether inertial measurement unit (IMU)-based real-time feedback gait for older inpatients immediately changes gait parameters. Seven older inpatients (mean age: 76.0 years) performed three types of 60-s gait trials with real-time feedback in each of the following categories: walking spontaneously (no feedback trial); focused on increasing the ankle plantarflexion angle during late stance (ankle trial); and focused on increasing the leg extension angle, which is defined by the location of the ankle joint relative to the hip joint in the sagittal plane, during late stance (leg trial). Tilt angles and accelerations of the pelvis and lower limb segments were measured using seven IMUs in pre- and post-feedback trials. To examine the immediate effects of IMU-based real-time feedback gait, multiple comparisons of the change in gait parameters were conducted. Real-time feedback increased gait speed, but it did not significantly differ in the control (*p* = 0.176), ankle (*p* = 0.237), and leg trials (*p* = 0.398). Step length was significantly increased after the ankle trial (*p* = 0.043, r = 0.77: large effect size). Regarding changes in gait kinematics, the leg trial increased leg extension angle compared to the no feedback trial (*p* = 0.048, r = 0.77: large effect size). IMU-based real-time feedback gait changed gait kinematics immediately, and this suggests the feasibility of a clinical application for overground gait training in older people.

## 1 Introduction

Decreased gait speed, propulsion, and range of motion of the lower extremities have been reported as typical changes in gait mechanics due to aging and various motor dysfunctions (Boyer et al., 2017). Such changes can lead to decreasing mobility and quality of life and an increased risk of adverse events (Abellan van Kan et al., 2009). Thus, it is important for older people to maintain gait speed as one of the determiners of gait ability.

Previous studies showed that gait speed did not increase by functional training such as resistance training alone (Kim et al., 2001; Ouellette et al., 2004). In order to improve gait ability, it is necessary to establish effective gait training procedures guided by individual gait characteristics (such as gait feedback training) (Franz et al., 2014; Schenck and Kesar, 2017; Genthe et al., 2018; Browne and Franz, 2019; Liu et al., 2020; Liu et al., 2021). Over the recent years, gait practice by using wearable sensors has been reported in clinical applications (Gordt et al., 2018; Hinton et al., 2023; Silva-Batista et al., 2023; Hinton et al., 2024), but its effect on increasing gait speed remains unclear. More specifically, previous studies had conducted gait feedback training using foot motion or foot pressure measured by a sensor attached to the dorsal foot and insole (Sungkarat et al., 2011; Byl et al., 2015). These reports have limited the target of gait feedback training, and it is necessary to establish methods that can be adapted to individual gait abnormalities in order to promote effective walking.

In order to apply an effective gait practice for older people in clinical settings, a system of gait feedback using multiple parameters was considered necessary. In clinical practice and cohort fields, we have analyzed human movement such as gait using inertial measurement units (IMUs) (Miyazaki et al., 2021a; Miyazaki et al., 2021b; Matsuzawa et al., 2021; Araki et al., 2023), and they are utilized in gait practice. However, there are few reports on the effect of gait feedback training performed in clinical practice (Hinton et al., 2023; Hinton et al., 2024), and more clinical data on this subject are needed. We have previously reported an IMU-based gait feedback system with real-time feedback of joint angles during overground gait, which showed increasing gait speed immediately with joint angle changes in young healthy adults (Miyazaki et al., 2023). There are various types of feedback (Sigrist et al., 2013), and our feedback system consists of extrinsic feedback, in which the knowledge of the result is provided by auditory stimulation. In addition, auditory stimulation is reported to be more effective than visual stimulation for dynamic postural control (Hasegawa et al., 2020). Our system uses auditory stimulation, can be implemented with a PC and IMU, making it easy to use in a clinical setting. Therefore, this system may be applicable to overground gait training for older people in clinical settings.

The purpose of this study was to examine whether IMU-based real-time feedback gait for older inpatients immediately changes gait parameters. The findings of this study offer fundamental data regarding effective gait practice for older inpatients in clinical settings. We hypothesized that IMU-based real-time feedback gait would lead to increased gait speed immediately, and specific changes in gait kinematics for each feedback target would also be observed.

## 2 Materials and methods

### 2.1 Participants

Seven older inpatients (mean age, 76.0 ± 7.1 years; including three women, four patients with orthopedic conditions, two patients post-stroke, and one patient with metabolic disease) who could walk several minutes without walking aids participated in this study (Table 1). The exclusion criteria were as follows: (1) lower-limb impairments such as pain that affected the measurement of gait and physical performance, (2) severe dementia, and (3) not consenting to participate in this study. Basic information, including disease, age, sex, height, and body mass index, was recorded. In addition, the five-times-sit-to-stand test (FTSS) was used as an indicator of physical performance. The FTSS involved standing up and sitting down five times from a sitting position, as quickly as possible, without pushing off (Mong et al., 2010). In the FTSS, well-trained assessors recorded the time taken to perform five consecutive chair-stands (timed to 0.1 s) from a seated position on a 45-cm-tall chair, with arms folded across the chest.

**TABLE 1:** Participants’ demographics.

	Disease	Age (y)	Sex	Height (m)	Weight (kg)	Comfortable gait speed (m/s)	FTSS (s)
1	TKA	68	M	1.59	84.6	1.33	6.05
2	TKA	73	F	1.55	63.3	0.83	13.51
3	LCS	71	F	1.44	67.1	0.95	8.95
4	VCF	71	F	1.56	54.0	1.03	13.6
5	CI	73	M	1.60	73.4	1.23	10.69
6	CI	88	M	1.49	45.1	0.79	8.44
7	DM	85	M	1.58	42.4	1.01	12.68
mean ± SD	-	75.6 ± 7.1	-	1.54 ± 0.05	61.4 ± 14.1	1.02 ± 0.18	10.56 ± 2.67

TKA, total knee arthroplasty; LCS, lumbar canal stenosis; VCF, vertical compression fracture; CI, cerebral infarction; DM, diabetes mellitus; M, male; F, female; FTSS, five-times-sit-to-stand test.

The study was approved by the Ethics Committee on Epidemiological Studies of Tarumizu Central Hospital (approval number: 20-8), and all participants provided written informed consent before participating in the study.

### 2.2 Feedback trials

**FIGURE 1:**
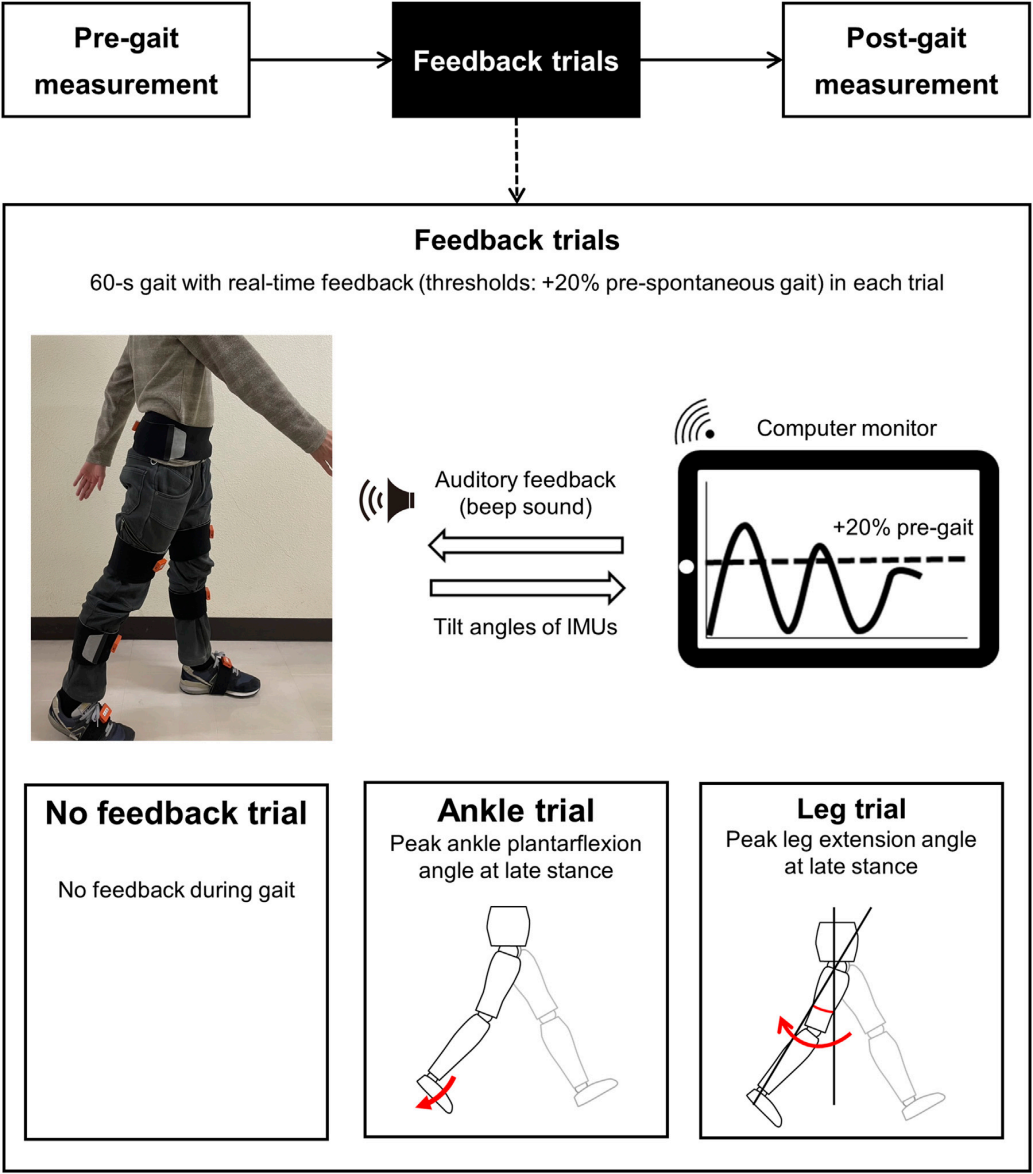
Experimental protocol of the IMU-based real-time feedback gait. At pre-gait (spontaneous gait) and post-gait (replicate gait without feedback) measurements, gait parameters were measured using IMUs. Pre-gait measurements also determined the threshold of feedback. During the feedback trials, participants modified gait in response to the beep sound when the participant’s current joint angle (solid line) reached the threshold angle (dot line). The threshold was set at a 20% increase in the peak values of each joint angle during spontaneous gait. IMUs: inertial measurement units.

As in previous studies (Miyazaki et al., 2023), gait parameters measured before and after the gait trials were compared to examine the immediate effects. During feedback trials, participants were instructed to modify their lower limb motion during gait under three types of feedback, and they walked on a 30-m walkway for 60 s in each trial (Liu et al., 2021; Miyazaki et al., 2023). Three feedback trials were performed (Figure 1): (i) a feedback trial without feedback (no feedback trial) and two feedback trials with real-time feedback during overground gait to (ii) increase the ankle plantarflexion angle during the late stance (ankle trial) and (iii) increase the leg extension angle, which is defined by the location of the ankle joint relative to the hip joint in the sagittal plane (Miyazaki et al., 2019), during the late stance (leg trial). Gait kinematics used as feedback targets were the ankle plantarflexion angle and leg extension angle at late stance. These parameters have been related to propulsion during gait (Hsiao et al., 2015a; Hsiao et al., 2015b; Browne and Franz, 2017; Browne and Franz, 2019), and they could be a feasible target for gait feedback training (Browne and Franz, 2019; Liu et al., 2020; Liu et al., 2021). Before each feedback trial, participants were explained the gait modification during each feedback trial by using verbal instructions and pictures. The details of the explanation were as follows: no feedback trial, “walk at your usual pace during this trial”; ankle trial, “push back the ground harder before you swing your leg so that it makes a beep sound during this trial”; and leg trial, “extend your leg farther backward before you swing your leg so that it makes a beep sound during this trial” (Miyazaki et al., 2023).

Before and after each feedback trial, participants walked along the 14-m walkway twice to measure gait parameters using IMUs (Figure 1). Spontaneous and replicate gait were measured pre- and post-feedback trials, and post-gait measurements were made without feedback. Each gait feedback trial consisted of one feedback trial and two gait measurement pre- and post-feedback trials (Figure 1), and they were randomly performed according to the Microsoft Excel Rand function. In addition, an approximate 2-min standing break interval was provided between each trial (Miyazaki et al., 2023). We measured the length of each patient’s right thigh and shank by using a measuring tape before the pre-gait measurement.

### 2.3 Methodology of the IMU-based real-time feedback gait

**FIGURE 2:**
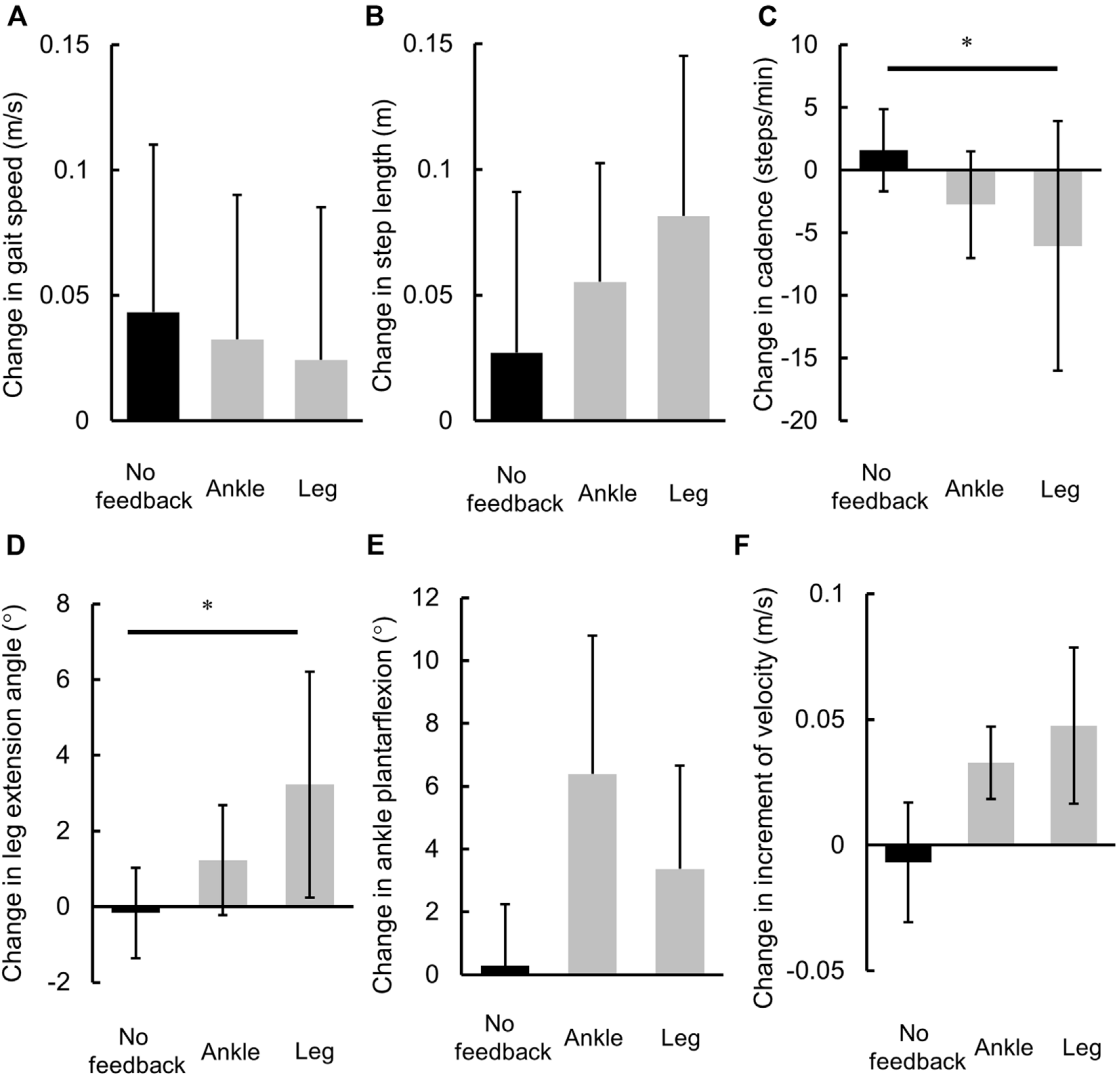
Comparisons of the changes in gait parameters. **(A)** Gait speed, **(B)** cadence, **(C)** step length, **(D)** maximum leg extension angle at late stance, **(E)** maximum ankle plantarflexion angle at late stance, and **(F)** increment of velocity at late stance. *: *p* < 0.05.

IMU-based real-time feedback gait was performed using a mobile PC (One-Mix3Pro, Tech-One Co. Ltd, Tokyo, Japan), and the joint angles calculated by IMUs were displayed on a PC (Figure 1) (Miyazaki et al., 2023). Gait parameters were measured using seven IMUs (MTw Awinda, Xsens, Enschede, NL), and the IMUs consisted of a 3D gyroscope, 3D accelerometer, and 3D magnetometer. The sampling frequency was 100 Hz. The 3-axis acceleration and tilt angles in a global coordinate system were obtained from the magnetic and inertial data using a Kalman filter on MT Manager software (4.7.2, Xsens, the Netherlands). The reliability of IMUs has been reported previously (Ferrari et al., 2010). Before gait measurements, IMUs were attached by elastic belts to the posterior sacrum, bilateral anterior thighs, shanks, and dorsal feet. For the dorsal feet, IMUs were fixed on their shoes (Figure 1). IMUs were also attached frontally and vertically against the frontal plane where possible, and they were calibrated so that the vertical direction of the coordinate system followed the direction of gravity during static standing (Miyazaki et al., 2019). The timing of the maximal posterior tilt angle of the sensor attached to each shank was used to determine the timing of initial contact (Revi et al., 2020). The PC screen displayed the joint angles calculated by the IMUs in real-time, and the threshold of the feedback was set at a 20% increase in the peak values of each joint angle during a spontaneous gait during the pre-feedback trial (Miyazaki et al., 2023). Participants were provided continuous real-time auditory feedback, and beep sounds were emitted when the participant’s current joint angle reached the threshold, during each feedback trial (Figure 2).

### 2.4 Data analysis

Low-pass filtering was performed on the joint angle, and acceleration data were measured using IMUs with a 10 Hz and 20 Hz cutoff frequency (Arumukhom Revi et al., 2021; Araki et al., 2024). For spatiotemporal parameters, cadence was calculated by identifying heel contact during the maximum posterior tilt angle of the sensor on the shank (Revi et al., 2020). Stride length and gait speed were also calculated based on the walking time measured by IMUs. The joint angles including the hip, knee, and ankle were calculated as relative Euler angles measured from IMUs fixed on the pelvis, thigh, shank, and foot segments (Araki et al., 2023; Miyazaki et al., 2023). In addition, the leg extension angle was determined based on the location of the ankle joint relative to the hip joint in the sagittal plane, estimated from the tilt angle matrix measured by IMUs and the vector of the thigh and shank segment coordinated by segment length (Miyazaki et al., 2019). Previous studies have confirmed the validity of using IMUs to determine these gait parameters (Miyazaki et al., 2019), and maximum ankle plantarflexion angle and leg extension angle during the late stance were calculated (Miyazaki et al., 2023). The increment of velocity was calculated using the anterior acceleration measured with the IMU fixed on the sacrum during late stance, which has also been reported as the association to the impulse of the anterior ground reaction force such as an indicator commonly used as propulsion force (Miyazaki et al., 2019). Thus, data processing was performed using the mathematical software MATLAB R2020a (Mathworks Inc., MA, United States).

### 2.5 Statistical analysis

The mean values of the variables determined for the bilateral lower extremities during 10 strides (five from the two gait measurements pre- and post-feedback trial, respectively) were used as the representative values. To confirm the normal distribution of the data, the Shapiro–Wilk test was conducted. To examine the immediate effects of the feedback trials (no feedback, ankle, and leg) on each gait parameter, the t-test and Mann–Whitney U-test were conducted. Then, to compare the change in gait parameters before and after the feedback trial, Friedman analysis was performed, and the Bonferroni method or Shaffer method was used to perform the multiple comparisons test. Calculations of r were performed to estimate the effect size of the group comparison. The effect size was classified into small (r = 0.10), medium (r = 0.30), and large (r > 0.50) effect sizes, as described previously (Cohen, 2013). All statistical analyses were performed using the software Statistical Package for the Social Sciences (SPSS 25, IBM, NY, United States), and the significance level was set at *p* = 0.05.

## 3 Results

### 3.1 Spatiotemporal gait parameters

In comparisons of pre- and post-feedback trials (Table 2), the step length was found to be significantly increased after the ankle trial (*p* = 0.043) and showed a tendency to increase after the leg trial (*p* = 0.063). Gait speed did not change after the control (*p* = 0.176), ankle (*p* = 0.237), and leg trials (*p* = 0.398). Cadence also did not significantly change after the control (*p* = 0.237), ankle (*p* = 0.176), and leg trials (*p* = 0.237).

**TABLE 2:** Individual changes in spatiotemporal and kinematic gait parameters after feedback trials.

	No feedback trial				Ankle trial				Leg trial			
	Pre	Post	Change (rate)	Effect Size	pre	Post	Change (rate)	Effect Size	pre	Post	Change (rate)	Effect Size
Spatiotemporal parameters												
Gait speed (m/s)	1.02 ± 0.2	1.03 ± 0.2	0.04 ± 0.1 (+4.2%)	0.51	1.05 ± 0.2	1.08 ± 0.2	0.03 ± 0.1 (+3.1%)	0.45	1.10 ± 0.2	1.12 ± 0.3	0.02 ± 0.1 (+2.2%)	0.32
Stride length (m)	1.03 ± 0.1	1.06 ± 0.2	0.03 ± 0.1 (+2.6%)	0.45	**1.06 ± 0.2**	**1.12 ± 0.1**	**0.06 ± 0.1 (+5.2%)**	0.77	1.10 ± 0.2	1.18 ± 0.2	0.08 ± 0.1 (+7.4%)	0.70
Cadence (steps/min)	118.1 ± 7.1	119.7 ± 6.0	1.60 ± 3.3 (+1.4%)	0.45	118.1 ± 7.9	115.3 ± 7.4	−2.75 ± 4.3 (−2.3%)	−0.51	119.8 ± 4.3	113.7 ± 12.6	−6.05 ± 10.0 (−5.0%)	−0.45
Kinematic parameters												
Ankle plantarflexion angle (°)	22.6 ± 6.8	22.8 ± 6.2	0.29 ± 2.0 (+1.3%)	0.06	**22.4 ± 6.6**	**28.8 ± 7.7**	**6.39 ± 4.4 (+28.5%)**	0.89	**20.9 ± 6.5**	**24.3 ± 9.2**	**3.37 ± 3.3 (+16.1%)**	0.83
Leg extension angle (°)	21.9 ± 2.3	21.7 ± 2.6	−0.17 ± 1.2 (−0.8%)	−0.06	22.2 ± 3.3	23.4 ± 3.8	1.23 ± 1.5 (+5.5%)	0.58	**23.3 ± 4.8**	**26.6 ± 4.9**	**3.23 ± 3.0 (+13.8%)**	0.77
Increment of velocity (m/s)	0.32 ± 0.02	0.31 ± 0.03	−0.01 ± 0.02 (−2.2%)	−0.26	**0.32 ± 0.04**	**0.36 ± 0.04**	**0.03 ± 0.01 (+10.2%)**	0.89	**0.33 ± 0.05**	**0.38 ± 0.05**	**0.05 ± 0.03 (+14.5%)**	0.89

Values are expressed as mean ± SD.

Bold font of values represents a significant difference at *p* < 0.05 between pre- and post-trial.

The effect size was classified as small (r = 0.10), medium (r = 0.30), and large (r > 0.50).

On comparison of the changes in spatiotemporal gait parameters, Gait speed and stride length did not differ between each feedback trial (Figures 2A, B). Cadence was found to differ significantly between each feedback trial, and it was decreased during the leg trial compared with the no feedback trial (*p* = 0.023, r = 0.54: large, Figure 2C).

### 3.2 Kinematic gait parameters

In comparisons of pre- and post-feedback trials (Table 2), ankle plantarflexion angle was found to be significantly increased after the ankle (*p* = 0.018) and leg trials (*p* = 0.028). The leg extension angle was significantly increased after the leg trial (*p* = 0.028). There was a significant increment of velocity after the ankle (*p* = 0.018) and leg trials (*p* = 0.018).

On comparison of the changes in kinematic gait parameters, leg extension angle was found to differ significantly between each feedback trial (*p* < 0.050), and it increased during the leg trial compared with the no feedback trial (*p* = 0.048, r = −0.49: medium, Figure 2D). The ankle plantarflexion angle and increment of velocity differed between each feedback trial (*p* = 0.066). The ankle plantarflexion angle showed a tendency to increase after the ankle trial compared with the no feedback trial (*p* = 0.098, r = −0.432: medium, Figure 2E), and there was a higher increment of velocity after the leg trial compared with the no feedback trial (*p* = 0.098, r = −0.432: medium, Figure 2F).

## 4 Discussion

In this study, we examined the immediate effects of IMU-based real-time feedback gait, focused on either the ankle or leg motion, during overground gait on gait kinematics in older inpatients. IMU-based real-time feedback gait in 60 s immediately changed spatiotemporal and kinematic gait parameters according to the feedback targets. Therefore, this study demonstrated the immediate effect of IMU-based real-time feedback gait focused on the motion of each joint, and it suggests the feasibility of its clinical application for overground gait training in older people.

IMU-based real-time feedback increased gait speed and showed a moderate effect size, but it was not significantly different for each feedback trial. In the ankle and leg trials, gait speed changed by a mean of 0.02–0.03 m/s, and a minimal detectable change in gait speed in community-dwelling older people (0.04–0.06 m/s) has not been observed (Perera et al., 2006). A previous report of older adults shows a similar trend, with no immediate increase in gait speed (mean change 0.08 m/s) after gait training in patients following a stroke (Hinton et al., 2023). In healthy participants using this gait training system, gait speed increased immediately after feedback trials (mean change 0.15–0.19 m/s) (Miyazaki et al., 2023), and these increases were close to or larger than 0.17 m/s, which is reported as the minimal detectable change in healthy participants (Meldrum et al., 2014). Of other spatio-temporal gait parameters, step length was increased after the ankle trial, showed a tendency to increase after the leg trial, and showed a large effect size. In addition, change in cadence was smaller in the leg trial than in the no feedback trial. Participants have experienced increased gait speed by changing gait strategies that alter either cadence or stride length or both (Howard et al., 2013; Baudendistel et al., 2021; Tateuchi et al., 2021). An immediate effect was observed in healthy adults, and a moderate to large effect size was shown for older inpatients. Thus, for older people in clinical settings, this gait training system may be effective in increasing gait speed through changing their gait strategy by considering intervention time, fatigue, and other factors.

In gait kinematic parameters, the leg extension angle was also significantly increased after the leg trial (mean change 3.2°), and change in the leg extension angle was larger in the leg trial than in the no feedback trial. The ankle plantarflexion angle was significantly increased after the ankle (mean change 6.4°) and leg trials (mean change 3.3°), and higher increment of velocity was observed after both trials. In addition, the ankle plantarflexion angle was significantly increased in the ankle trial compared to the no feedback trial; meanwhile, there was a higher increment of velocity in the leg trial compared to the no feedback trial. These parameters also showed a moderate or greater effect size. Sufficient forward movement of the center of gravity ensured an increase in leg extension angle (Bowden et al., 2006; Balasubramanian et al., 2007; Turns et al., 2007), which also leads to an increase in the propulsion force (Hsiao et al., 2015a; b; Hsiao et al., 2016; Browne and Franz, 2017). Similar to the leg extension angle, the increment of velocity during the late stance is an indicator of the propulsion force (Miyazaki et al., 2019), and ankle plantarflexion angle also contributes to increase in step length and propulsion force during gait (Hsiao et al., 2015a; Hsiao et al., 2015b; Zelik and Adamczyk, 2016; Browne and Franz, 2017). In addition, the measurement error of the leg extension angle is reported as 1.4°–1.9° (Miyazaki et al., 2019), and the minimal detectable change is also reported as 3.8° for the leg extension angle (Kesar et al., 2011) and 2.6° for the ankle plantarflexion angle (Molina-Rueda et al., 2021); changes in these parameters in the leg and ankle trials of the current study were close to or larger than these figures. These gait kinematics during the late stance would be akin to an increase in push-off power, and we facilitated their immediate change using real-time feedback. Therefore, this IMU-based real-time feedback gait is capable of immediately changing gait parameters related to forward propulsion, giving it the potential to improve walking efficiency in older people in clinical settings.

### 4.1 Potential implications for effective gait practice for older inpatients in clinical settings

Although the sample was small, we were able to implement the protocol for older inpatients. This study did not show a similar immediate effect to that reported for healthy young participants (Miyazaki et al., 2023), but we believe that the current IMU-based real-time feedback gait system has potential for clinical application. The strength of this system is that multiple parameters can be selected, so it is necessary to consider which parameters are most informative, and further study is needed to realize gait practice using the most appropriate feedback target for individuals. Decreasing ankle push-off and propulsion force at the late stance have been reported as gait parameters that change with aging (Boyer et al., 2017) along with dependence on the proximal joint compared with healthy young adults (DeVita and Hortobagyi, 2000; Hortobagyi et al., 2016; Kuhman et al., 2018; Conway and Franz, 2020). In older adults at risk for mobility, disability showed a faster preferred gait speed and physical function in the group with increased stride length compared with the group with increased cadence (Baudendistel et al., 2021). Conversely, another report demonstrates the relationship between ankle power and forward shift of the center of gravity during gait in older people (Sloot et al., 2021). In this study, the mean values of participants were 1.02 m/s for gait speed and 10.56 s for FTSS. In addition, this study especially showed immediate changes in gait kinematics during ankle trials. Therefore, the ankle motion might be a suitable target of IMU-based real-time feedback gait for efficiently increasing gait speed in older people without physical function decline, who did not meet the criteria for physical function decline in sarcopenia (<1.0 m/s for gait speed and/or 12.0 s for FTSS) (Chen et al., 2020).

### 4.2 Limitations

Our study had several limitations. First, this study examined only the immediate effect and was not able to examine long-term intervention effects. Second, this system used only auditory feedback, making it difficult to set the threshold between the lower and upper limits. In previous studies, gait feedback training using audio and visual feedback was performed using treadmills and monitors that fitted within the optimal range of thresholds (Schenck and Kesar, 2017; Liu et al., 2020; Liu et al., 2021). Comparisons with other feedback methods such as auditory and vibratory stimulations are also needed. Third, fatigue after each trial was not assessed, and it is unclear whether the intensity of gait feedback was appropriate. Fourth, the small sample size may have increased the variability of outcome measures. Finally, physical function was measured only by FTSS. In this study, participants did not meet the criteria for physical function decline in sarcopenia (Chen et al., 2020). In clinical settings, it is anticipated that this gait training will be implemented for inpatients with poorer physical function. More detailed and varied measurements of physical function, such as individual muscle strength and balance ability, are needed. It is necessary to accumulate several cases to verify the effectiveness of gait training under controlled conditions of disease and physical function. Since the latter systems are not feasible in a clinical setting, we believe that our gait feedback system is more likely to be used in clinical settings. Despite these limitations, this study showed that an immediate change in gait kinematics was observed, and it provides evidence of effective overground gait training for older people in clinical settings.

## 5 Conclusion

In this study, IMU-based real-time feedback gait immediately changed gait parameters according to the types of each joint motion at late stance during overground gait in older inpatients. This IMU-based real-time feedback gait system also allows multiple gait parameters to be selected, which could lead to effective overground gait training using feedback targets appropriate for each inpatient in clinical settings. To achieve effective gait practice in clinical settings for older inpatients, further study is needed to clarify the long-term effects of IMU-based real-time feedback gait on gait parameters and the appropriate target of gait practice for each individual.

## Data Availability

The raw data supporting the conclusions of this article will be made available by the authors, without undue reservation.
